# Left-sided fibrothorax: a sequela of chronic tubercular empyema

**DOI:** 10.11604/pamj.2024.47.126.42736

**Published:** 2024-03-21

**Authors:** Gaurang Aurangabadkar, Sumer Choudhary

**Affiliations:** 1 Department of Respiratory Medicine, Datta Meghe Medical College, Nagpur, Datta Meghe Institute of Higher Education and Research (DMIHER) Deemed University (DU), Sawangi (Meghe), Wardha, India

**Keywords:** Fibrothorax, tuberculosis, empyema

## Image in medicine

A 70-year-old male patient presented to the respiratory medicine outpatient department (OPD) with chief complaints of left-sided chest pain and dyspnea on exertion for the last 2 years. The patient also had complaints of cough with occasional mucoid expectoration for the last 2 months. A detailed history revealed a history of left-sided tubercular empyema in 2018, in which the patient had taken anti-tubercular treatment for 6 months, and was informed that he did not go to the pulmonologist for regular follow-up as advised. A chest X-ray posteroanterior (PA) view was done, which revealed the presence of left-sided pleural thickening along with left-sided lung volume loss (red arrow) and compensatory hyperinflation of the right lung. Fibrothorax is defined as pleural fibrosis that may occur secondary to an inflammatory reaction due to connective tissue diseases, asbestos exposure-related pleural diseases, or drug reaction, with the commonest cause being tubercular empyema, especially in a country with a high tuberculosis burden such as India. In this patient, given the history of chronic tubercular empyema, this chest X-ray image highlights the long-standing and often debilitating sequelae of tuberculosis. Therefore, we wish to emphasize the importance of the comprehensive and multi-disciplinary approach that is needed in the treatment of tubercular empyema with the active involvement of a thoracic surgeon, to prevent the development of such complications in patients with pleural tuberculosis.

**Figure 1:**
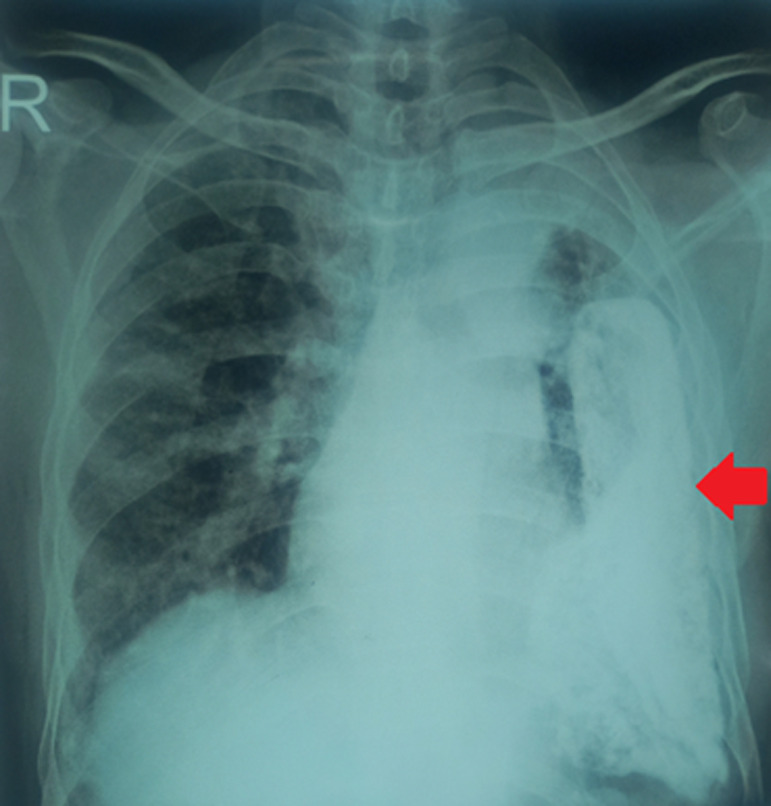
chest X-ray posteroanterior view of the patient showing left-sided fibrothorax (red arrow) due to chronic tubercular empyema; right lung showing compensatory hyperinflation

